# Psychometric properties of the Children’s Response Styles Questionnaire in a Hong Kong Chinese community sample

**DOI:** 10.1186/s12955-017-0774-x

**Published:** 2017-10-10

**Authors:** Barbara Chuen Yee Lo, Yue Zhao, Yim Chi Ho, Terry Kit-fong Au

**Affiliations:** 1 The University of Hong Kong, Hong Kong, Hong Kong, Special Administrative Region of China; 2 Po Leung Kuk, Hong Kong, Hong Kong, Special Administrative Region of China

**Keywords:** Children’s coping styles: Rumination, Distraction, Problem solving, Children's Response Styles Questionnaire, item response theory

## Abstract

**Background:**

The Children’s Response Styles Questionnaire (CRSQ) is a widely-adopted inventory that assesses response styles in youths. It is useful in examining how coping styles (particularly rumination) may relate to depressive vulnerability in youths. Despite its utility, little is known about its applicability in non-Western cultures and CRSQ has not been evaluated using current psychometric methods including item response theory (IRT). The present study assessed the properties using IRT methods in a Chinese youth sample.

**Methods:**

Students in Grades 4-6 were recruited from seven public primary schools in Hong Kong, and a total of 581 children (280 boys and 301 girls) between 8 and 14 years of age participated in the study. A Chinese version of CRSQ was administered to them in groups at school after receiving written parental consent as well as students’ assent.

**Results:**

Confirmatory factor analysis revealed a two-factor structure that was comparable to that identified in Western samples, namely, the rumination and distraction/problem-solving subscales. IRT analysis suggested that items varied in levels of item discrimination and item severity, and in precision/usefulness for assessing the underlying latent trait levels. Test information analysis indicated that rumination subscale was more useful than the distraction and problem-solving subscale in assessing the latent trait over a broader range of levels. For gender-based Differential Item Functioning (DIF) analysis, item 1 “When I am sad, I think about how alone I feel” was found to exhibit higher discriminating power for girls than boys.

**Conclusions:**

The study presents the first attempt to examine CRSQ item properties using IRT analysis and supports its validity beyond the Western cultures. The factor structure of CRSQ was found to be comparable to the West in our Chinese sample. Differential Item Functioning (DIF) evaluation suggested all but one item in the rumination subscale of the CRSQ apply equally well to both boys and girls.

## Background

One can respond to a mood change (e.g. a sudden onset of low mood) using different coping strategies, ranging from a total distraction strategy to constantly focusing on the issue (i.e. rumination) in an attempt to deal with or gain insight into the mood change [1, 2]. According to the Response Styles Theory (RST), the choice and the combination of response styles may affect subsequent mood regulation abilities and information processing [1, 2]. One’s preferred response style may well be a trait-like, stable characteristic [2], arising from modeling from parents, social or problem-solving skills, sex-role expectations, genetics, physiological reactivity and so forth.

Research has suggested that repetitive thinking in the form of rumination is a characteristic symptom of psychiatric disorders such as depression [2–4]. People who ruminate about negative experience often tend to experience more intense dysphoric mood [3–7], report impaired concentration and problem-solving abilities [8–10], and have more accessible negative memories [11, 12].

Amongst children, the ruminative response style predicts depressive symptoms concurrently and months or even years later (e.g. [13–15]). By contrast, distraction and problem solving seem to predict reduced depression risks (e.g. [15, 16]). Empirically, the RST applies equally well to children and adolescents. In addition, previous literature has robustly indicated that internalizing symptoms occurring particularly during the critical period of early- to mid-adolescence significantly predict risks and duration of emotional problems later in life [17]. Assessing coping styles early in the developmental trajectory therefore is of paramount importance to risk identification and promoting healthy adolescent development.

To identify ruminative response style early in life, an appropriate and valid measure is crucial. Although adult forms of coping style inventories can be applied to youth populations, many argue that the construction of a new scale specifically tailor-made to assessing the phenomenology observed among youths is preferable [18]. The Children’s Response Styles Questionnaire (CRSQ) was developed in this context [13, 19, 20]. In comparison to other assessment tools that also target youths’ coping styles (e.g. the Children’s Coping Strategies Scale (CCSS) [21]; the Perseverative Thinking Questionnaire–Child Version (PTQ-C [22]), CRSQ items were derived specifically based on the RST [5]. This unique approach has the potential to make the CRSQ sensitive to youths’ coping in response to depressive mood, as compared to assessing coping response to stressful events in general [20].

Since its first publication in 2000, the CRSQ has become one of the most widely adopted measures for assessing children’s coping styles [23]. However, most of its research and applications were conducted in Western cultures [24, 25], and studies on its validation and utility in Eastern cultures are rather limited [23, 26]. In China, rapid economic growth and urbanization over the past few decades have raised significant concerns over children and adolescents’ mental health and wellbeing, particularly concerning the academic and other kinds of competition and stress they may have experienced. Some studies indicated that between 10 and 30% of Chinese youths may have experienced mental health problems in various forms [27], with youth depression prevalence on the rise [28, 29]. It is hence useful to validate a relatively quick, simple and inexpensive tool, such as the CRSQ, for assessing risk predictors of Chinese children’s depression problems. In the past, cultural variance in depressive instruments has been reported [30, 31], and one would wonder if the factor structure for CRSQ identified in Western cultures would hold in Eastern cultures as well. Traditionally, Eastern cultures such as the Chinese culture emphasize collectivism, interpersonal harmony and social responsibility. Such cultural values can in theory encourage more socioemotion-focused coping strategies such as rumination as opposed to problem solving [32]. Importantly, maladaptive coping such as rumination, in contrast with adaptive coping such as problem solving, has been hypothesized to increase depressive symptoms among Chinese youths [33].

In its original format [19], CRSQ was developed with three subscales: rumination, distraction and problem-solving subscale. The rumination subscale assesses an individual’s tendency to self-focus on depressed mood and its consequences. The distraction response subscale assesses the tendency to divert attention away from sad mood. The problem-solving subscale assesses the tendency to use practical strategies to alter negative mood. In later revision, a two-factor version was validated such that the rumination factor was kept but the distraction and the problem-solving items were combined into a second factor [13]. The CRSQ, with good psychometric properties, has been widely adopted to assess how different response styles (particularly in the case of ruminative style) relate to stress reactivity and depressive vulnerability in youths. For example, Stewart et al. [34] employed an experimental stress test and found that depressed teenage participants with high trait rumination showed delayed post-stressor physiological (cortisol) recovery, while those with high trait distraction and problem solving exhibited more rapid recovery. Abela and colleagues [35, 36], adopting a multi-wave longitudinal design using self-report measures, also observed that a youth’s ruminative tendency as indicated by CRSQ moderated the relationship between occurrence of negative/stressful events and subsequent risks for developing depressive symptoms in early adolescence.

In spite of being included in a number of applications, the CRSQ has not been evaluated in terms of item-level psychometric properties using current measurement theory. Note that different questionnaire items may tap different levels of severity of the underlying latent trait (i.e. response styles), and hence can have different discriminating abilities among individuals with varied levels of the latent trait. Given gender differences reported on childhood depression and coping styles [1, 7], the item property may also operate differently across different groups (e.g. different genders) of youths. To address these issues, analysis based on item response theory (IRT; [37]) can be a good strategy.

Relative to Classical Test Theory (CTT), IRT has the following advantages. First, IRT provides estimates on individual item’s discrimination and severity that are sample independent [37]. Examining item properties (e.g. item discrimination and item severity) of the CRSQ with IRT helps elucidate the psychometric properties of the CRSQ and deepen our understanding of the RST. Second, IRT can estimate the underlying latent trait level for each individual based on his/her endorsement on each item, rather than getting only total symptom counts from CTT-based analysis. Individuals who endorse the same response style (e.g. ruminative style) on a more severe item or a less severe item are more likely to have different levels of the latent trait. Third, IRT provides item information on how well a particular item contributes to the assessment of the latent trait along a continuum ranging from low to high level. Items with higher information values, denoting more precision for measuring the level of the latent trait, are more useful than those items with lower information values. Furthermore, test information (i.e. sum of the item information of all items) reveals how well an instrument as a whole contributes to the assessment of the latent trait along its continuum. Last but not least, IRT can access differential item functioning (DIF) to identify items that function differently on subgroups such as gender groups, after controlling for the latent trait measured by the CRSQ. Specifically, Abela, Aydin, and Auerbach [13] found no significant difference between boys and girls in the rumination subscale of the CRSQ on the basis of the observed scores. However, it remains unclear whether a certain item may be more discriminating and sensitive to more severe level of the underlying latent trait for one gender group than the other.

In summary, little research has evaluated the validity of CRSQ in non-Western cultures to date (e.g. [23]), even though cultural factors can influence coping processes [38]. To our knowledge, this is the first attempt to evaluate the psychometric properties of CRSQ in Chinese. The present study set out to address the following questions. First, is the factor structure of the CRSQ instrument derived from the Western population valid and useful in the Chinese context? Second, what are the item properties (item discrimination and item severity) of individual CRSQ items? Third, does the CRSQ function equally well between boys and girls in terms of item discrimination and severity?

## Methods

### Data collection

Information on the study and parent/guardian consent forms were distributed to Grades 4-6 students of seven public primary schools in Hong Kong between March 2009 to September 2012. Only students who were able to read and write Chinese were included in the study. A final sample of 581 children (280 boys and 301 girls), with ages between 8 to 14 years (M = 10.6, SD = 1.1), was secured with written parental consent as well as students’ assent before data collection. This sample size was estimated to be adequate for performing factor analysis and IRT analysis based on the 25-item CRSQ [39]. In each school, the Chinese version of the CRSQ questionnaire was administered by a research assistant in group format, which the participants took less than 20 min to complete. The questionnaire was filled in anonymously, and participating children were advised to ask the research assistant if they had any questions about the items. Each questionnaire was checked if there was any missing items at the time of submission. Participants were thanked at the end of the session.

### Measure

#### Children's Response Styles Questionnaire (CRSQ)

The CRSQ is a self-report questionnaire consisting of 25 items to assess children’s coping styles for sad moods. For each item, children were asked to rate on a 4-point scale (“almost never”, “sometimes”, “often”, and “almost always”) on how frequently they would adopt a certain coping strategy when they feel sad (e.g. "When I am sad, I think about how alone I feel"). In its original form, the CRSQ was grouped into three subscales: the ruminative response subscale, the distracting response subscale, and the problem-solving subscale [19]. In a later revision of the CRSQ, Abela and colleagues [13] examined the factor structure of the proposed subscales and found a good fit for a two-factor model. Their findings showed a strong correlation between the distraction and problem-solving subscales which loaded onto the same factor. Based on this factor structure, the CRSQ was refined and subsequently divided into two subscales: a rumination factor (13 items; scores ranging from 0 to 39) and a distraction/problem-solving factor (12 items; scores ranging from 0 to 36). The total score in each subscale was calculated by adding the sum of the respective item scores. Higher mean scores on each subscale imply a greater tendency to engage in the particular response style associated with the subscale. The CRSQ was originally developed and tested in English [13, 19, 20] and later translated and tested in Turkish [23]. The instrument has evidence for structural validity based on confirmatory factor analysis [13, 23], internal consistency [13, 15, 35, 40] and test-retest reliability [20]. Evidence for construct validity includes correlations of the rumination scale scores with perseverative thinking in children [22] and self-critical perfectionism tendency in children [41], and both rumination and distraction/problem solving scores with depression [36].

The Chinese version of CRSQ was developed by our research team. The original CRSQ was translated from English into Chinese by one translator and then back-translating by another translator independently. The two translators were university graduates proficient in both English and Chinese. The translated and back-translated questionnaires were then compared and evaluated by the first and last authors to finalize the wordings of this Chinese version of CRSQ.

### Data analysis

The following statistical analyses were conducted: 1) Exploratory factor analysis and confirmatory factor analysis were performed to examine the factor structure of the CRSQ. 2) An IRT analysis was carried out to evaluate the psychometric properties of the questionnaire on the item level, and 3) A IRT-based differential item functioning (DIF) analysis was conducted to detect whether the CRSQ items function applied equally well to boys and girls.

To examine the factor structure of the CRSQ in the Chinese context, exploratory factor analysis (EFA) was first performed, followed by confirmatory factor analysis (CFA). Since the CRSQ has not been validated in the Chinese context, considering the cultural variance, it was necessary to investigate its factor structure starting with an exploratory approach. The numbers of dominant factors were determined based on the point at which the eigenvalues change from a rapid and decelerating decline to a gradually flat slope in the scree plot [42–44]. EFA was performed in SPSS 20.0 [45] using varimax rotation, assuming orthogonal constructs between the factors based on previous findings [13]. Using a factor loading of 0.30 as the cut-off [46], items with a factor loading smaller than 0.30 or having loadings on multiple factors were eliminated. Next, CFA was performed based on polychoric correlation matrix using diagonally weighted least squares in LISREL 8.8 [47].

IRT item calibration was carried out using the graded response model (GRM; [48]) in PARSCALE [49] for two main considerations: (a) classical item analysis suggested that item discrimination parameters were needed for this dataset, and (b) GRM is one of the widely used IRT models in clinical measurement where the item responses are polytomous and ordered (see e.g. [50, 51]). The degree of IRT model-data fit was evaluated by using both statistical tests of significance and graphical displays, as suggested by Swaminathan, Hambleton, & Rogers [52]. The likelihood ratio goodness-of-fit statistic *G* ^2^ [53] provided by PARSCALE was used for examining model fit. For the potentially misfit items suggested by *G* ^2^, they were further evaluated in graphical representation by examining the discrepancies between observed response proportions and IRT based predicted response proportions along the *θ* continuum.

Given gender differences for childhood depression and coping styles [1, 5], differential item functioning (DIF) analysis was conducted to examine whether the CRSQ items function equally for boys and girls after controlling for the latent trait measured by the CRSQ. The IRT-LR analysis was performed in IRTLRDIF [54]. Iterative purification procedure was applied for identifying the anchor set, which was free of DIF and used to link the two comparison groups on a common metric [50, 55]. The adjustment of Benjamini-Hochberg (B-H) procedure was adopted for controlling the Type I error rate in multiple comparisons and for determining the statistical significance of identifying DIF [50].

## Results

### Factor structure

To examine the factor structure of the CRSQ in the Chinese context, EFA was first performed. The eigenvalues greater than 1.0 [42] were respectively 4.73, 4.05, 1.43, 1.15 and 1.05, collectively accounting for 49.63% of the total variance. Two dominant factors emerged and they together explained 35.14% of the total variance. In the two-factor solution, items 1, 3, 5, 7, 9, 11, 13, 19, 21 and 25 loaded onto one factor which reflected rumination with factor loading values ranging from .39 to .71 and items 2, 4, 6, 8, 10, 12, 14, 16, 18, 20, 22 and 24 onto the other factor which reflected distraction and problem-solving with factor loading values ranging from .22 to .71. Using factor loading of 0.30 as a cut-off, items 15, 17 and 23 loaded onto both factors with similar factor loading values, and they were thus removed. In addition, items 4 and 14 had low item-total correlations (with values below .30) and were hence eliminated from the distraction and problem-solving subscale.

Next, a two-factor CFA model was employed. The same structure obtained in EFA was applied to the two-factor CFA solution. Item factor loadings were all positive, ranging from 0.36 to 0.73. The goodness-of-fit indices of the two-factor CFA (CFI = 0.92, GFI = 0.90, RMSEA = 0.07, SRMR = 0.08) suggested an acceptable fit. The correlation between the two latent factors was .02, suggesting that the two subscales were nearly independent. Therefore, IRT and DIF analyses were subsequently conducted on the individual subscales.

Tables 1 and 2 show the descriptive statistics and item-total correlations for the final 20-item version of the CRSQ-Chinese. Cronbach’s alpha was 0.82 for the rumination scale (CRSQ-R), and 0.81 for the distraction/problem-solving scale (CRSQ-DPS).

**Table 1:** Item Content, Response Frequencies, Item Statistics and DIF Statistics for the Rumination Subscale of the CRSQ (*N* = 581)

Item	Observed Response Frequencies (%)^a^				Classic Item Statistics^b^			Factor Loading	Item Parameter Estimates^c^				Fit Index (*G* ^2^)^d^	Gender DIF (χ^2^)^e^	
	0	1	2	3	*M*	*SD*	*r*		*a*	*b*_*1*_	*b*_*2*_	*b*_*3*_		*a-* DIF	*b-* DIF
1. When I am sad, I think about how alone I feel	28.57	54.22	11.19	6.02	0.95	0.79	0.55	0.50	1.67	−0.82	1.33	2.29	66.01	7.1*	7.3
3. When I am sad, I go away by myself and think about why I feel this way	32.01	34.42	22.03	11.53	1.13	0.99	0.46	0.49	1.08	−0.88	0.74	2.23	96.24*	−	−
5. When I am sad, I think: “I’m ruining everything”	51.98	27.54	12.05	8.43	0.77	0.96	0.51	0.55	1.43	0.07	1.28	2.18	59.30	−	−
7. When I am sad, I think about how sad I feel	39.93	35.97	11.88	12.22	0.96	1.00	0.54	0.62	1.56	−0.40	1.00	1.72	52.30	−	−
9. When I am sad, I go some place alone to think about my feelings	42.17	29.78	15.32	12.74	0.99	1.04	0.42	0.46	0.95	−0.44	1.09	2.30	56.56	−	−
11. When I am sad, I think about how angry I am with myself	51.98	28.4	11.88	7.75	0.75	0.94	0.57	0.60	1.78	0.05	1.17	2.02	38.75	−	−
13. When I am sad, I think about other times when I have felt sad	35.11	30.98	17.56	16.35	1.15	1.08	0.59	0.73	1.86	−0.52	0.55	1.33	70.95	−	−
19. When I am sad, I think: “I’m disappointing my friends, family, or teachers”	48.19	29.26	13.25	9.29	0.84	0.98	0.38	0.40	0.93	−0.12	1.51	2.78	50.74	−	−
21. When I am sad, I think about all my failures, faults, and mistakes	33.91	37.52	14.63	13.94	1.09	1.02	0.59	0.67	1.80	−0.58	0.76	1.50	41.56	−	−
25. When I am sad, I think about how I don’t feel like doing anything	34.42	35.11	14.46	16.01	1.12	1.06	0.43	0.50	1.04	−0.76	0.94	1.89	57.33	−	−

^a^Response score categories contain: 0 = “Almost Never”, 1 = “Sometimes”, 2 = “Often”, and 3 = “Almost Always”

^b^ *M* = mean. *SD* = standard deviation. *r* = item-total correlation

^c^ *a* = item discrimination parameter estimates, *b*_1_, *b*_2_, *b*_3_ = item severity parameter estimates

^d^Fit index: likelihood ratio goodness-of-fit statistic *G* ^2^ provided by PARSCALE. A nonsignificant result (Benjamini-Hochberg adjusted overall alpha level of .05) is an indicator of adequate model fit

^e^Gender differential item functioning (DIF): tested using the likelihood ratio-based significance test under the IRT framework (IRT-LR) in an iterative purification procedure for identifying DIF free anchor set. “−” indicates the anchor items which are free of differential item functioning (DIF)

^*^ *p* < Benjamini-Hochberg adjusted overall alpha level of .05

**Table 2:** Item Content, Response Frequencies, Item Statistics and DIF Statistics for the Distraction and Problem-Solving Subscale of the CRSQ (N = 581)

Item	Observed Response Frequencies (%)^a^				Classic Item Statistics^b^			Factor Loading	Item Parameter Estimates^c^				Fit Index (*G* ^2^)^d^	Gender DIF (χ^2^)^e^	
	0	1	2	3	Mean	SD	*r*		*a*	*b*_*1*_	*b*_*2*_	*b*_*3*_		*a-* DIF	*b-* DIF
2. When I am sad, I help someone else with something so I don’t think about my problem	39.59	40.96	15.83	3.61	0.83	0.82	0.45	0.41	1.18	−0.47	1.47	3.28	106.85*	−	−
6. When I am sad, I go to my favorite place to get my mind off my feelings	42.17	29.43	16.87	11.53	0.98	1.03	0.38	0.43	0.99	−0.39	1.11	2.39	58.03	−	−
8. When I am sad, I spend a lot of time on my schoolwork	48.36	34.08	13.08	4.48	0.74	0.85	0.38	0.36	0.97	−0.09	1.88	3.59	57.36	5.6	0.6
10. When I am sad, I do something I enjoy	15.32	39.24	26.51	18.93	1.49	0.97	0.45	0.49	1.22	−1.76	0.22	1.52	70.95*	−	−
12. When I am sad, I do something fun with a friend	24.27	29.95	27.02	18.76	1.40	1.05	0.56	0.66	1.63	−1.00	0.17	1.29	48.56	−	−
16. When I am sad, I ask a friend, parent, or teacher to help me solve my problem	32.19	34.77	16.52	16.52	1.17	1.06	0.52	0.63	1.46	−0.71	0.66	1.49	35.37	0.7	9.7
18. When I am sad, I try to find something good in the situation or something I learned	32.7	39.41	18.07	9.81	1.05	0.95	0.59	0.63	1.77	−0.63	0.81	1.82	57.84	−	−
20. When I am sad, I talk it out with someone who I think can help me feel better	26.51	28.74	24.1	20.65	1.39	1.09	0.52	0.64	1.43	−0.98	0.20	1.27	56.58	0.8	10.7
22. When I am sad, I think of a way to make my problem better	20.83	42.51	25.99	10.67	1.27	0.91	0.54	0.55	1.48	−1.25	0.53	1.93	49.12	−	−
24. When I am sad, I remind myself that this feeling will go away	25.65	34.94	20.48	18.93	1.33	1.06	0.42	0.50	1.04	−1.23	0.52	1.67	45.92	−	−

^a^Response score categories contain: 0 = “Almost Never”, 1 = “Sometimes”, 2 = “Often”, and 3 = “Almost Always”

^b^ *M* = mean. *SD* = standard deviation. *r* = item-total correlation

^c^ *a* = item discrimination parameter estimates, *b*_1_, *b*_2_, *b*_3_ = item severity parameter estimates

^d^Fit index: likelihood ratio goodness-of-fit statistic *G* ^2^ provided by PARSCALE. A nonsignificant result (Benjamini-Hochberg adjusted overall alpha level of .05) is an indicator of adequate model fit

^e^Gender differential item functioning (DIF): tested using the likelihood ratio-based significance test under the IRT framework (IRT-LR) in an iterative purification procedure for identifying DIF free anchor set. “−” indicates the anchor items which are free of differential item functioning (DIF)

^*^ *p* < Benjamini-Hochberg adjusted overall alpha level of .05

### IRT analysis

*Dimensionality checking.* In the rumination subscale, essential unidimensionality was supported because the first eigenvalue (3.88) was substantially greater than the second one (0.98) and the ratio of the first to second eigenvalue were approximately 4, generally accepted as evidence for unidimensionality [56]. In addition, the goodness-of-fit indices from the single-factor CFA model (CFI = .97, GFI = .97, RMSEA = .06, SRMR = .04) suggested a good fit. Item factor loadings were all positive, ranging from 0.40 to 0.73 (Table 1). In the distraction and problem-solving subscale, similarly, the first eigenvalue (3.69) was substantially greater than the second one (0.996) thereby supporting unidimensional assumption. Moreover, the goodness-of-fit indices from the single-factor CFA model (CFI = .94, GFI = .95, RMSEA = .07, SRMR = .05) indicated a reasonable fit. Item factor loadings were all positive, ranging from 0.36 to 0.64 (Table 2).

### IRT calibration and goodness-of-fit assessment

In the rumination subscale, the item discrimination parameters ranged from 0.93 to 1.86 (Table 1). The highest discriminating item was item 13 “When I am sad, I think about other times when I have felt sad” (*a* = 1.86), and the lowest discriminating item was item 19 “When I am sad, I think: I’m disappointing my friends, family, or teachers” (*a* = 0.93). In terms of item severity (Table 1), item 11 “When I am sad, I think about how angry I am with myself” (*b*_1_ = 0.05, *b*_2_ = 1.17, *b*_3_ = 2.02), item 5 “When I am sad, I think: I’m ruining everything” (*b*_1_ = 0.07, *b*_2_ = 1.28, *b*_3_ = 2.18) and item 19 “When I am sad, I think: I’m disappointing my friends, family, or teachers” (*b*_1_ = −0.12, *b*_2_ = 1.51, *b*_3_ = 2.78) emerged at the higher levels of severity. Similarly, their observed item means were also lower than the other CRSQ-R items. Items 3 “When I am sad, I go away by myself and think about why I feel this way” (*b*_1_ = −0.88, *b*_2_ = 0.74, *b*_3_ = 2.23) and item 25 “When I am sad, I think about how I don’t feel like doing anything” (*b*_1_ = −0.76, *b*_2_ = 0.94, *b*_3_ = 1.89) reflected lower levels of severity in the CRSQ-R subscale.

**Fig. 1:**
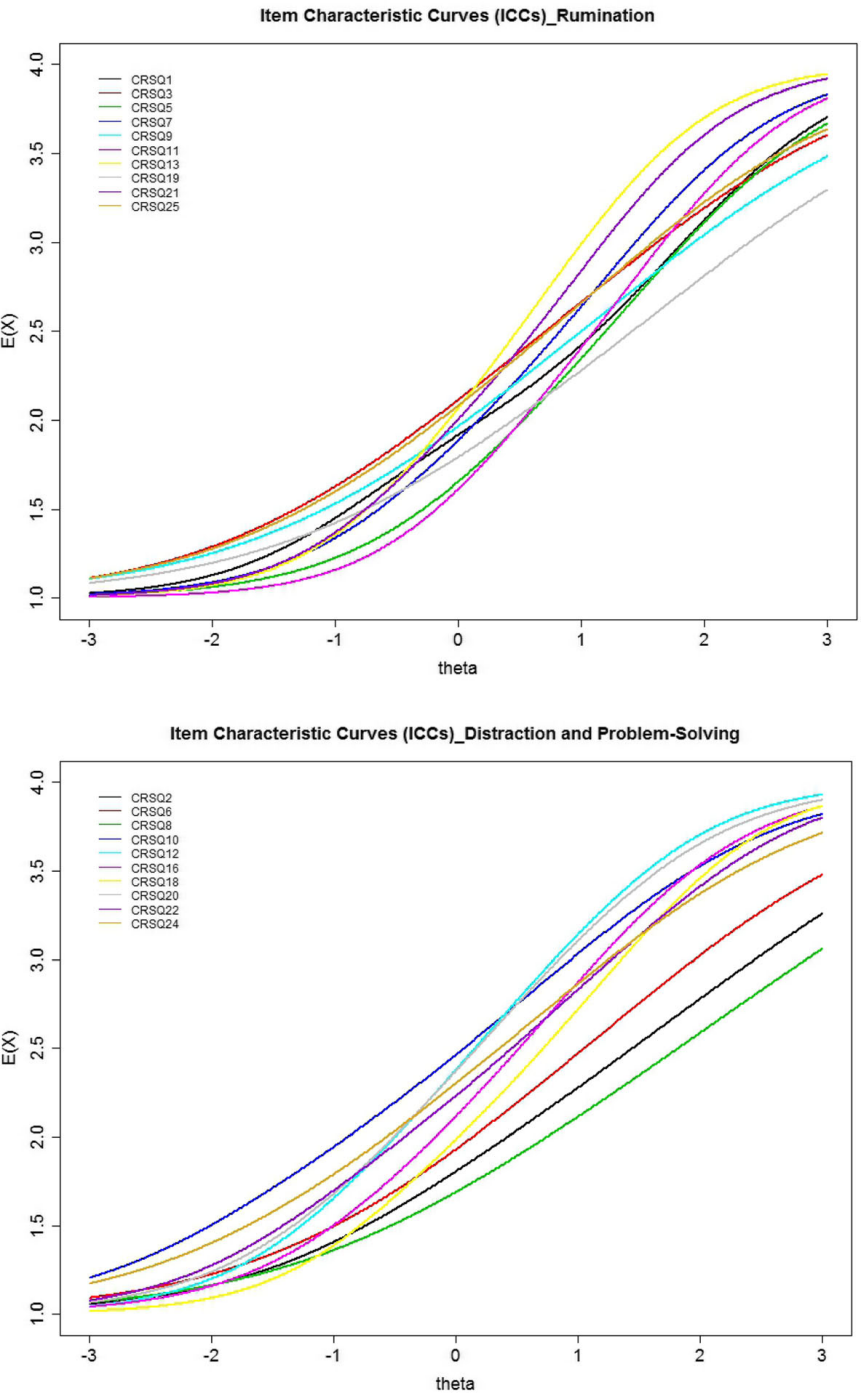
Item characteristic curves (ICCs) for the subscales of rumination and distraction/ problem-solving of the CRSQ. Note: the raw response categories 0 to 3 were recoded as 1-4 respectively in the IRT analysis. Response score categories contain: 1 = “Almost Never”, 2 = “Sometimes”, 3 = “Often”, and 4 = “Almost always”

**Fig. 2:**
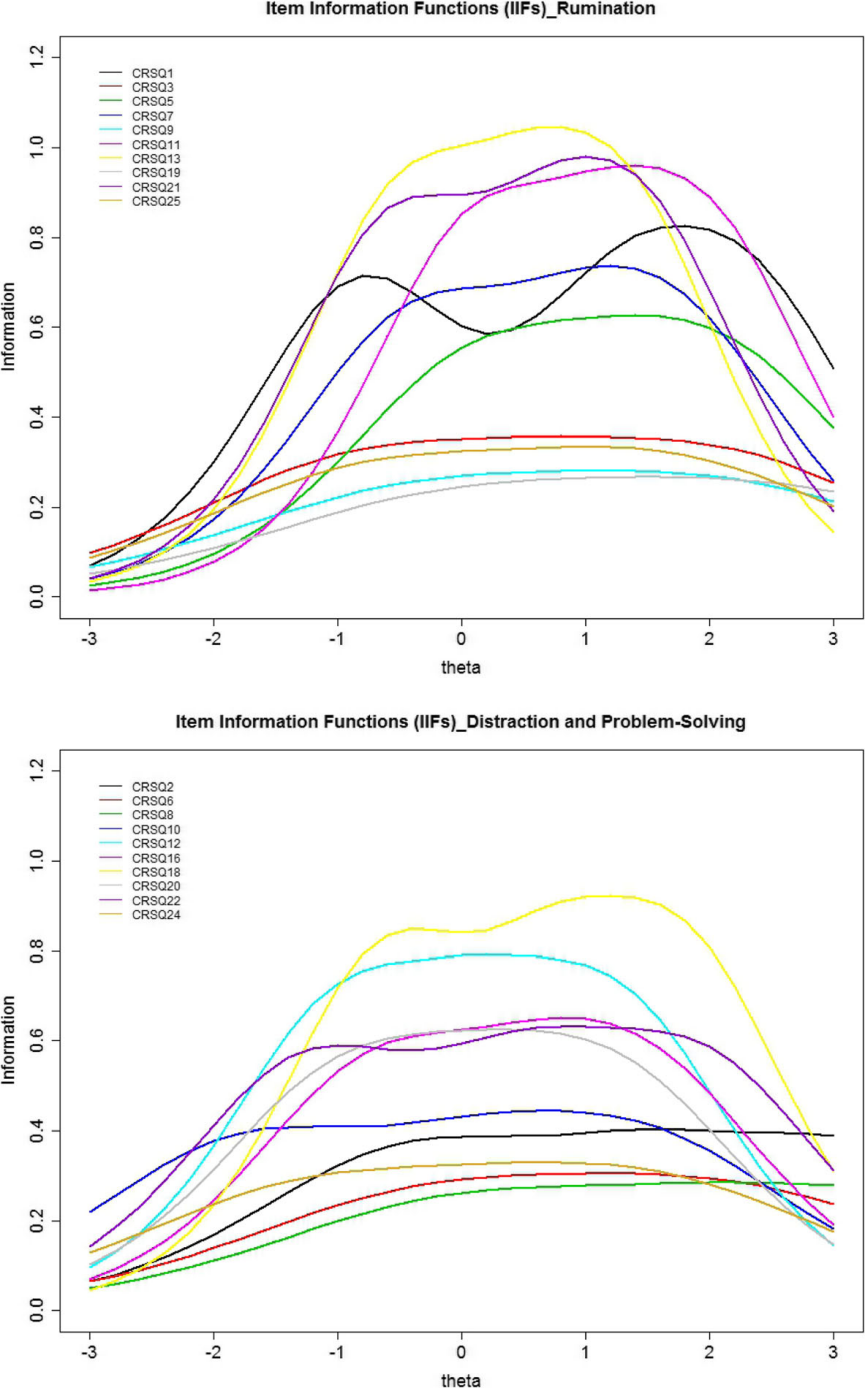
Item information functions (IIFs) for the subscales of rumination and distraction/ problem-solving of the CRSQ

As can be seen in the item characteristic curves (ICCs) and item information functions (IIFs) respectively in Figs. 1 and 2, among the 10 CRSQ-R items, item 13 “When I am sad, I think about other times when I have felt sad” clearly offered the most information along a wide range of *θ* roughly from −1 to +1.5. Items 11 “When I am sad, I think about how angry I am with myself” and 21 “When I am sad, I think about all my failures, faults, and mistakes” offered greater potential for discriminating among respondents than most of the remaining items; in particular, item 11 was more useful for differentiating upper *θ* levels (approximately between +1.5 and +2.4), whereas item 21 provided more information for respondents with moderate *θ* levels (approximately between −0.5 and +1.2). Item 19 “When I am sad, I think: ‘I’m disappointing my friends, family, or teachers’”, item 9 “When I am sad, I go someplace alone to think about my feelings”, item 25 “When I am sad, I think about how I don’t feel like doing anything” and item 3 “When I am sad, I go away by myself and think about why I feel this way” − the low discriminating items − exhibited a flatter ICC, adding very little to the psychometric quality of the CRSQ-R because the power of these items to discriminate respondents with higher from lower levels was minimal.

In the distraction and problem-solving subscale, the item discrimination parameters ranged from 0.97 to 1.77 (Table 2). The highest discriminating item was item 18 “When I am sad, I try to find something good in the situation or something I learned” (*a* = 1.77), and the lowest discriminating item was item 8 “When I am sad, I spend a lot of time on my schoolwork” (*a* = 0.97). In terms of item severity, as shown in Table 2, item 8 “When I am sad, I spend a lot of time on my schoolwork” (*b*_1_ = −0.09, *b*_2_ = 1.88, *b*_3_ = 3.59) and item 2 “When I am sad, I help someone else with something so I don’t think about my problem” (*b*_1_ = −0.47, *b*_2_ = 1.47, *b*_3_ = 3.28) reported higher levels of severity (or strength of the latent trait for adopting the distraction or problem-solving coping style). Consistently, less than 5% of respondents rated “almost always” for both items, as shown in the observed response frequencies in Table 2. Given the community sample in the study, even those near the higher end of latent trait levels (e.g. *θ* = 3) would reach only an expected item score around the third response category (“often”), and very few cases would endorse the fourth response category (“almost always”), as shown in Fig. 2. Among those with lower level of severity in the CRSQ-DPS subscale were item 10 “When I am sad, I do something I enjoy” (*b*_1_ = −1.76, *b*_2_ = 0.22, *b*_3_ = 1.52), item 12 “When I am sad, I do something fun with a friend” (*b*_1_ = −1.00, *b*_2_ = 0.17, *b*_3_ = 1.29) and item 20 “When I am sad, I talk it out with someone who I think can help me feel better” (*b*_1_ = −0.98, *b*_2_ = 0.20, *b*_3_ = 1.27).

Among the 10 CRSQ-DPS items, item 18 “When I am sad, I try to find something good in the situation or something I learned” provided more information than the other nine items along the wide range of *θ* continuum approximately between −1 and +2.5. Items 8, 6, 24 and 2 had lower discriminating parameter estimates and thus offered less information than the other items. Between items 16 “When I am sad, I ask a friend, parent, or teacher to help me solve my problem” and 22 “When I am sad, I think of a way to make my problem better” with similar *a* parameters, item 16 was slightly more useful than item 22 in discriminating among the respondents with moderate latent trait levels (*θ* between around −0.5 and +1), whereas item 22 offered slightly more useful information in discriminating among the respondents in the higher end and the lower end of the *θ* continuum.

**Fig. 3:**
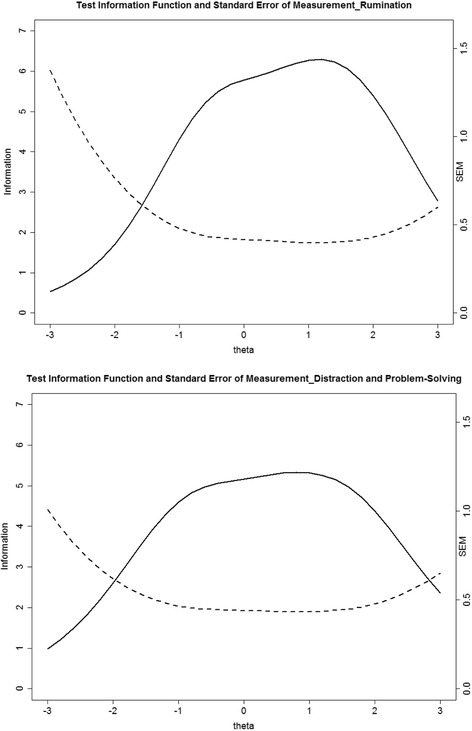
Test Information Function (TIF) and Standard Error of Measurement (SEM) Curve for the subscales of rumination and distraction/problem-solving separately. Note: Upper panel: Solid lines; left y-axis = total information aggregated across all items assessing rumination along the latent trait (*θ*, theta) ranging from −3 to 3. Dashed lines; right y-axis = standard error of measurement for the scale of rumination along the latent trait (*θ*, theta) ranging from −3 to 3. Lower panel: Solid lines; left y-axis = total information aggregated across all items assessing distraction/problem-solving along the latent trait (*θ*, theta) ranging from −3 to 3. Dashed lines; right y-axis = standard error of measurement for the scale of distraction/problem-solving along the latent trait (*θ*, theta) ranging from −3 to 3

In addition to the item level, test information and standard errors of measurement along the *θ* continuum were produced in Fig. 3. The CRSQ-R subscale and CRSQ-DPS subscale offered the most useful information at the *θ* value around 1.2 and 1 respectively (approximately one standard deviation above the mean). Specifically, the CRSQ-R was more informative in assessing respondents along the *θ* continuum approximately between −1 and +2 (between one standard deviation below the mean and two standard divisions above the mean), and CRSQ-DPS was more informative in assessing respondents along the *θ* continuum approximately between −0.5 and +0.5 (between half standard deviation below the mean and half standard deviation above the mean). The performance of test information suggested that the both subscales were informative in assessing a broad range of the latent trait, whereas the CRSQ-R offered great potential in assessing the higher level of the latent trait, and it hence can be particularly useful for screening high risk individuals with elevated latent trait levels.

Furthermore, the degree of IRT model-data fit was evaluated by using both statistical tests of significance and graphical displays. Using *G* ^2^ provided by PARSCALE, items 2, 3 and 10 were detected as potentially misfit items, and they were further evaluated in graphical representation. The confidence bands of predicted item performances generally covered the observed item performances, except a few outliers in the first, second and fourth score category curves. We thus considered acceptable fit for the three items.

#### Differential item functioning analysis

Using the iterative purification procedure, among the 20 CRSQ items, the DIF-free anchor set consisted of nine items (i.e., items 3, 5, 7, 9, 11, 13, 19, 21 and 25) in the CRSQ-R subscale and seven items (i.e., items 2, 6, 10, 12, 18, 22 and 24) in the CRSQ-DPS subscale, and the remaining four items were candidate items that were used for testing DIF. The χ^2^ values for the nested model comparison tests of *a*-DIF and *b*-DIF for the four candidate items were shown in Tables 1 and 2. Any CRSQ-R item with χ^2^ associated probability less than B-H adjusted overall alpha level of .05 was flagged as DIF.

As can be seen in Tables 1 and 2, gender DIF was identified on the item discrimination parameters (*a*’s) on item 1 “When I am sad, I think about how alone I feel” in favor of girls. In other words, the item exhibited higher discriminating power for girls than boys. Among all studied items, no significant differences on the item severity parameters (*b*’s) across gender were detected, suggesting that the 20 CRSQ items function equally between boys and girls in terms of item severity.

## Discussion

### Validity of the Children's Response Styles Questionnaire in the Chinese context

For factor structure, the present study yielded similar findings as those documented with a Western sample by Abela, Aydin & Auerbach [13]. Importantly, the two-factor structure identified was comparable across ours and Abela et al.’s [13], implying that the CRSQ has a similar format in Chinese school age children as in some Western cultures. In contrast to the original form of 25-item CRSQ in English [19], a 20-item Chinese CRSQ emerged from item elimination in our data analyses, with 10 items remaining in the rumination subscale and 10 items in the distraction and problem-solving subscale. The correlation between the two factors (*r* = .02) suggested that the constructs were nearly orthogonal in the Chinese context, which coincides with the previous findings identified in the Western samples [13, 19]. On a related note, the removal of items loading onto both factors helped to reduce the correlation between the two factors. Also, the Chinese version not only has fewer items, but item 8 “When I am sad, I spend a lot of time on my schoolwork” and item 6“When I am sad, I go to my favorite place to get my mind off my feelings” that had been deleted from the earlier 21-item version in Abela et al.’s study [13], stayed in the distraction and problem-solving subscale in the Chinese version. Specifically, these two items relate to academic performance or personal space, and this may reflect some cultural difference in which achieving well academically and getting away from a crowd could be perceived as “adaptive” problem-solving or distraction skills among Chinese youths. For internal consistency, the Cronbach’s alpha was .82 for the CRSQ-R subscale and .81 for the CRSQ-DPS subscale for this 20-item Chinese CRSQ. Similar moderate levels of internal consistency (alphas = 0.82 and 0.79 for the CRSQ rumination and distraction/problem solving subscales respectively) were reported in previous studies in the Western context [13]. The figures in both subscales suggested adequate internal consistency, with .70 as a commonly accepted minimum reliability estimate in psychological and educational measurement [57].

### Item properties of the Children's Response Styles Questionnaire in the Chinese context

Our study was the first to utilize IRT methods to evaluate the item properties of the CRSQ. Our IRT analysis suggested that some symptoms reflected higher levels of severity than others. In the rumination scale, items such as “ruining everything” and “disappointing my friends, family, or teachers” emerged at the higher levels of severity, whereas items such as “go away by myself” and “don’t feel like doing anything” reflected lower levels of severity. Importantly, the more severe items are consistent with negative evaluation phenomenon commonly observed in depressive rumination among Chinese youths [18]. In Confucian societies, performing in the best interest of the group is highly emphasized [58]. With a narrowing of focused attention resulted from chronic rumination, the risk of a person feeling worthless due to failure to perform socially-oriented duties increases [18], leading to a sense of sustained helplessness and hopelessness, which can further fuel a vicious cycle of ruminative response.

In the distraction and problem-solving subscale, items such as “spending a lot of time on schoolwork” and “helping someone else with something” reflected higher levels of severity, implying that youngsters endorsing these items may be better equipped with problem solving abilities or resources to endorse action-oriented distraction strategies. Overall, the findings on the various levels of severity, may contribute to the clinical utility of the CRSQ. Individuals endorsing the same ratings on the more severe items and on less severe items are more likely to have different levels of the latent trait.

Results from the IRT analysis also suggested that some symptoms are stronger discrimination indicators than others. For instance, the item “thinking about other times when I have felt sad” in the rumination subscale and the item “trying to find something good in the situation or something I learned” in the problem-solving/distraction subscale were found to have higher discriminating power. Clinicians may do well to pay special attentions to these discriminating symptoms because they are particularly robust in differentiating varied levels of the latent trait ranging from low to high. Interestingly, some items such as “disappointing my friends, family, or teachers” in the rumination subscale and “spending a lot of time on schoolwork” in the problem-solving/distraction subscale showed the lowest discriminating power. We again suggest that this may be related to collectivistic cultural reasons. To consider expectations from others and achieve well academically (i.e. studying for lengthy periods) are commonly highlighted as the primary “responsibility” for youths in Asian Confucian societies. These imperative cultural values have been internalized and deeply-rooted in their mindset, thus making the suggested items comparatively insensitive and less discriminating in differentiating response styles in such societies.

Regarding the test information, both subscales offered the most useful information at the latent trait level approximately one standard deviation above the mean (on the *θ* scale). The rumination subscale was more useful in assessing the latent trait at a broad range of levels from about one standard deviation below the mean to about two standard deviations above the mean on the *θ* scale. The distraction and problem-solving subscale was more useful in assessing the latent trait around half standard deviation below and above the mean on the *θ* scale. In other words, both subscales may be informative and useful in assessing the latent trait for community samples of Asian school-age children where the expected average level is not at the extreme end. It is noteworthy that the rumination subscale assessed the latent trait over a wider range of levels, compared to the distraction and problem-solving subscale, rendering the former particularly useful for screening high risk individuals with elevated latent trait levels. High ruminative tendency had been widely considered a significant risk factor for psychopathology, given its strong association with and predictive power for depressive symptoms [14, 15].

DIF analysis showed that the scale items generally apply equally well to boys and girls, except that item 1 “When I am sad, I think about how alone I feel” exhibited higher discriminating power for girls. Understandably, girls may be more susceptible to evaluating themselves by comparing how acceptable they are by others or how much social ties/ resources they have. Girls’, more so than boys’, self-concepts generally depend on interpersonal aspects [59]. Quite plausibly, this gender difference is exacerbated in collectivistic societies, as social desirability is even more deeply valued. Notwithstanding this significant gender effect in the first item, all other items in CRSQ seem to work equally well for boys and girls in assessing the ruminative trait.

### Limitations and future studies

We are mindful of several limitations of the present study. First, we relied on self-report measures in assessing psychometric properties of the Children’s Response Styles Questionnaire. Future studies using information from other sources or structured diagnostic interviews to corroborate self-reports will be helpful. Second, our study only recruited local children between ages 9 and 14 years. It remained to be seen whether the factor structure observed could be generalized to other age groups. Specifically, an important ongoing debate focuses on the age at which children develop stable response styles in light of their cognitive development ([19, 20]; see [60] for review). Further studies are needed to explore if the factor structure uncovered in this study will also turn out to be valid for younger or older children. Such information can have significant implications for the clinical utility of this user-friendly questionnaire as an effective assessment tool for early identification of children at risk for depression.

## Conclusions

Our major findings are as follows. First, our factor analyses suggested that this Chinese and briefer version of CRSQ had a factor structure similar to that in the West [13]. Second, item properties from the IRT analysis suggested that the 20 items in the trimmed CRSQ reflected various levels of item discrimination and item severity, and contributed in various degrees of precision/usefulness to the assessment of the underlying latent trait levels. Third, findings from test information indicated that the rumination subscale was useful in assessing the latent trait over a broader range of levels, compared to the distraction and problem-solving subscale. In particular, the rumination subscale may be particularly useful for screening high risk individuals. Last, DIF evaluation suggested that all but one item in the rumination subscale of the CRSQ applied equally well to boys and girls.

## Abbreviations

B-H: Benjamini-Hochberg
CFA: Confirmatory factor analysis
CFI: Comparative fit index
CRSQ: Children’s response styles questionnaire
CTT: Classical test theory
DIF: Differential item functioning
DPS: Distraction and problem-solving subscale
EFA: Exploratory factor analysis
GFI: Goodness-of-fit index
GRM: Graded response model
ICC: Item characteristic curve
IIF: Item information function
IRT: Item response theory
LD: Local dependence
LR: Likelihood ratio
R: Rumination subscale
RMSEA: Root mean square error of approximation
SD: Standard deviation
SEM: Standard error of measurement
SRMR: Standardized root mean square residual
TIF: Test information function
